# Serum Levels of Calcium, Phosphate, and Vitamin D and Incident Arrhythmias: A Prospective Cohort Study of 348,094 UK Biobank Participants

**DOI:** 10.3390/nu17243895

**Published:** 2025-12-12

**Authors:** Pei Qin, Frederick K. Ho, Carlos A. Celis-Morales, Jill P. Pell

**Affiliations:** 1 School of Health and Wellbeing, University of Glasgow, Glasgow G12 8TB, UK; frederick.ho@glasgow.ac.uk (F.K.H.); jill.pell@glasgow.ac.uk (J.P.P.); 2 Department of Behavioural Science and Health, University College London, 1-19 Torrington Place, London WC1E 7HB, UK; 3 School of Cardiovascular and Metabolic Health, University of Glasgow, Glasgow G12 8TB, UK; carlos.celis@glasgow.ac.uk; 4 Human Performance Lab, Education, Physical Activity and Health Research Unit, University Católica del Maule, Talca 3460000, Chile; 5 Centro de Investigación en Medicina de Altura (CEIMA), Universidad Arturo Prat, Iquique 1110939, Chile

**Keywords:** serum calcium, phosphorus, vitamin D, arrhythmias, atrial fibrillation, bradyarrhythmia, ventricular arrhythmias, dose–response

## Abstract

**Background/Objectives:** Evidence of associations between serum calcium, phosphate, and vitamin D concentrations and development of arrhythmias is limited and inconsistent. This study aimed to investigate the quantification and characterization of the dose–response relationship between serum mineral levels and arrhythmia subtypes in a general population cohort. **Methods:** We included 348,094 UK Biobank participants without prevalent cardiovascular disease or arrhythmias in whom serum calcium, phosphate, and vitamin D concentrations were available. Serum calcium and phosphate levels were multiplied to derive calcium–phosphate product. Incident outcomes were all arrhythmias and subtypes: AF, other (non-AF) arrhythmias, bradyarrhythmia, and ventricular arrhythmias. Cox proportional hazards regression models were conducted. **Results:** Compared with the lowest quartile, participants in the highest quartile of serum calcium had a significantly lower risk of all arrhythmias (hazard ratio [HR] 0.92, 95% confidence interval [CI], 0.88–0.95), particularly AF (0.89, 0.85–0.93). Negative associations were found between serum vitamin D and arrhythmias, especially ventricular arrhythmias (HR 0.68, 95% CI 0.57–0.81). Higher serum phosphate and calcium–phosphate product were associated with higher risk of all outcomes. Restricted cubic splines revealed nonlinear associations for calcium and vitamin D but linear associations for phosphate and calcium–phosphate product. The associations were not modified by kidney function. **Conclusions:** Lower calcium and vitamin D concentrations and higher serum phosphate and calcium–phosphate product were associated with increased risk of arrhythmias and presented a dose–response manner. These findings may indicate that maintaining optimal serum calcium, phosphate, and vitamin D may be important for reducing arrhythmic risk, emphasizing the need for targeted monitoring and management, particularly in high-risk populations.

## 1. Introduction

Arrhythmias are associated with a number of long-term risks, including cardiac arrest, stroke, and heart failure. The prevalence of the most common form of arrhythmia, atrial fibrillation (AF), has increased approximately 30-fold over the past three decades, reaching 59.7 million globally in 2019 [1]. Hence, it is important to identify and address modifiable risk factors for the development of arrhythmias.

Calcium is the most abundant mineral in the human body and plays a vital role in many biological processes, such as intracellular signaling, circulatory homeostasis, muscle contraction, and nervous system function. A meta-analysis showed a positive association between serum calcium and risk of coronary heart disease and myocardial infarction [2]; however, another study found that the relationship may be U-shaped rather than linear [3]. A meta-analysis of cohort studies and randomized controlled trials showed an elevated risk of cardiovascular events among participants taking calcium supplements [4]. Although intracellular calcium has a critical role in the regulation of cardiovascular physiology, observational studies of the association between serum calcium concentrations and arrhythmias are few in number [5,6] and focused on patients in intensive care units or undergoing cardiac surgery [6]. Moreover, investigation is lacking into the dose–response relationship between serum calcium and arrhythmias.

Phosphorus is an essential mineral in the human body, playing a critical role in numerous physiological functions, such as building cell membranes, signal transduction, bone health, and energy metabolism [7]. Most phosphorus in the human body is present as phosphate, bound to proteins in the blood, with the remaining 30% circulating as inorganic phosphate [8]. Elevated phosphorus concentrations are associated with higher risk of carotid atherosclerosis and cardiovascular morbidity and mortality [9,10]. Conversely, hypophosphatemia has been proposed as a cause of new cardiac arrhythmias in critically ill patients [11,12]; however, few studies have investigated the association between serum phosphate and cardiac arrhythmias in the general population [13], and there is a paucity of information on the dose–response relationship.

Vitamin D is essential to bone health because of its role in regulating calcium and phosphate homeostasis. Studies have shown associations between both serum vitamin D and vitamin D supplementation and CVD [14,15] but studies of the association between serum vitamin D and AF are few in number and have reported conflicting findings [16]. No study, to our knowledge, has explored the dose–response relationship or the associations between vitamin D and risk of total arrhythmias or other arrhythmia subtypes.

In summary, observational studies on the associations of serum calcium, phosphorus, and vitamin D and arrhythmias are few in number, and existing studies had limitations such as the small sample size and focusing on disease populations or one subtype of arrhythmias. There remain several knowledge gaps in whether associations exist, their magnitude, and the shape of relationships. Therefore, we aimed to investigate the associations between serum calcium, phosphorus, calcium–phosphate product, and vitamin D and incident arrhythmias, overall and by subtype: atrial fibrillation, other arrhythmias (non-AF arrhythmias), bradyarrhythmias, and ventricular arrhythmias. The relationships were further explored, as was the potential modifying effect of kidney function.

## 2. Materials and Methods

### 2.1. Study Population

UK Biobank was a prospective cohort study that recruited approximately half a million participants from the general population aged 37–73 years between 2006 and 2010. Baseline data were collected at 22 assessment centers across England, Wales, and Scotland, using touchscreen questionnaires, verbal interviews, physical and medical measurements, and biological sampling [17]. UK Biobank was approved by the Northwest Multi-center Research Ethics Committee in the UK (Ref: 11/NW/0382) and was conducted in accordance with the Declaration of Helsinki. Written informed consent was provided by all participants. More information about the UK Biobank protocol is available online (http://www.ukbiobank.ac.uk).

**Figure 1:**
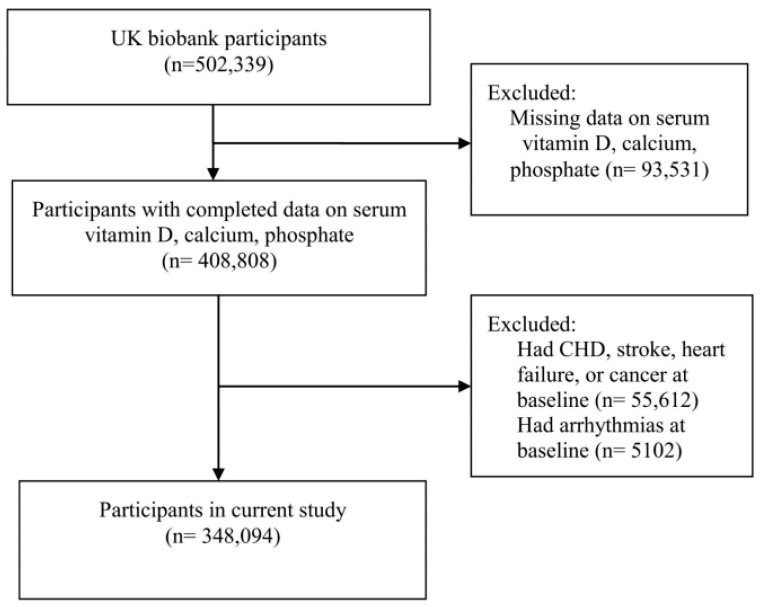
Flowchart of participant inclusion.

The exclusion criteria of the present study were missing baseline measurements of serum calcium, phosphorus, or vitamin D (*n* = 93,531) and having coronary artery disease, stroke, heart failure, or cancer (*n* = 55,612) or arrhythmias (*n* = 5102) at baseline, ascertained from self-reported physician diagnosis at enrollment or retrospective linkage to electronic health records with relevant ICD codes. After applying these criteria, 348,094 participants were included in the analysis (Figure 1).

### 2.2. Measurement of Exposures

In UK Biobank, mineral metabolism markers were measured at the central laboratory using serum samples collected at baseline (2006–2010). Serum calcium (mmol/L) and phosphate (mmol/L) concentrations were determined on the Beckman Coulter AU5800 platform (Beckman Coulter AU5800, Beckman Coulter UK Ltd., Amersham, UK), using a colorimetric method and phosphomolybdate complex analysis, respectively. Serum vitamin D (nmol/L) concentration was measured by chemiluminescence immunoassay on the DiaSorin Liaison XL platform (DiaSorin Liaison XL, Diasorin S.p.A, Saluggia, Vercelli, Piedmont, Italy). The calcium–phosphate product was calculated by multiplying values of serum calcium and serum phosphate.

### 2.3. Ascertainment of Outcomes

The outcomes of interest in this study were incident total arrhythmias and arrhythmia subtypes: AF, other arrhythmias (all arrhythmias excluding AF), bradyarrhythmia, and ventricular arrhythmias. The outcomes were ascertained from hospital admissions and death records based on International Statistical Classification of Diseases and Related Health Problems, Tenth Revision (ICD-10) codes (Table S1).

### 2.4. Ascertainment of Covariates

The covariates were all measured at baseline and were selected based on the existing literature [18]. Sociodemographic variables included age, sex, ethnicity, and the Townsend deprivation index [19]. Lifestyle factors included smoking status (never, previous, current), alcohol consumption (units per week), physical activity (low, moderate, high), total sedentary time (hours per day), fruit and vegetable intake (portions per day), red meat intake (0, 0–2, >2 times/week), processed meat intake (0, 0–2, >2 times/week), and sleep duration (1–6, 7–8, ≥9 h). Physical examinations, biochemical measurements, and history of disease were also included: body mass index (BMI, kg/m^2^), waist circumference (WC, cm), systolic blood pressure (SBP, mmHg), total cholesterol (mmol/L), high-density lipoprotein (HDL) cholesterol (mmol/L), glycated hemoglobin (HbA1c [mmol/mol]), estimated glomerular filtration rate (eGFR), and the number of long-term conditions (0, 1, ≥2). Medications included antihypertensive medication, cholesterol-lowering medication, aspirin, and insulin.

The average number of units of alcohol consumed weekly for participants was estimated using the questions regarding whether they consumed alcohol, frequency of consumption, and the typical weekly/monthly consumption of different types of alcoholic drinks [20]. Intake of the total processed and each type of unprocessed (i.e., unprocessed beef, lamb/mutton, and pork) red meats, self-reported at baseline using a food frequency questionnaire, was transformed into ordinal categories: 0, 0.5, 1, 2–4, 5–6, and ≥7 times/week); then these were summed to derive total consumption of processed red meat (range 0–7 times/week) and unprocessed red meat (range 0–21 times/week). Total sedentary time was calculated from the sum of television or computer screen watching and leisure time driving. eGFR was derived using the European Kidney Function Consortium sex- and race-free equation based on serum creatinine [21]. The number of long-term conditions was calculated from a list of 43 long-term conditions self-reported at baseline (Table S2) [22]. Further details of these measurements can be found in the UK Biobank online protocol (https://www.ukbiobank.ac.uk/).

### 2.5. Statistical Analyses

Baseline characteristics were reported as mean and standard deviation (SD) or median and interquartile range (IQR) for continuous variables with normal or non-normal distributions, respectively, and number (percentage) for categorical variables. Analysis of variance (ANOVA), Mann–Whitney U, and χ^2^ tests were used to test the differences in baseline characteristics across groups.

Cox proportional hazard regression models were performed to estimate the hazard ratios (HRs) and their 95% confidence intervals (CIs) for the associations between serum calcium, phosphate, calcium–phosphate product, and vitamin D concentrations and incident arrhythmias. The exposures were modelled as quartiles, then per 1 SD increase. Proportional hazard assumptions were tested using Schoenfeld residuals tests and log–log survival plots. Variation inflation factors (VIFs) were applied to examine the presence of multicollinearity among the variables. Three models were used: model 1 adjusted for age, sex, Townsend deprivation index (continuous), and ethnicity; model 2 additionally adjusted for smoking status, alcohol consumption, sleep duration, fruit and vegetable intake, processed meat intake, red meat intake, physical activity level, and total sedentary time; and model 3 additionally adjusted for HDL-cholesterol concentration, total cholesterol concentration, SBP, BMI, WC, HbA1c, eGFR, antihypertensive medication use, cholesterol-lowering medication, aspirin, insulin, and number of long-term conditions. A multivariable-adjusted restricted cubic spline with four knots was used to clarify the dose–response relationship. Subgroup analyses by the median value of eGFR were conducted. We tested interaction terms between decreased kidney function and the different serum minerals to assess whether the associations were modified by decreased kidney function.

The main models were run using complete case analyses. Sensitivity analyses were performed using multiple imputation with chained equations to derive missing covariate data, assuming the data were conditionally missing at random. Landmark analyses were also performed excluding participants with incident arrhythmias in the first two years of follow-up (*n* = 3090). Moreover, we corrected for multiple testing using Bonferroni correction (*p* < 0.0025; based on α = 0.05 divided by the test numbers across 5 outcomes and 4 exposures). All analyses were conducted using R, version 4.3.2 (R Foundation for Statistical Computing, Vienna, Austria) statistical packages. Two-tailed *p*-values < 0.05 were considered to indicate statistical significance.

## 3. Results

Of 348,094 participants in the study population, 32,674 (9.4%) developed arrhythmias over a median 13.7 year follow-up: 21,230 (6.1%) incident AF, 17,070 (4.9%) other arrhythmias, 5007 (1.4%) bradyarrhythmia, and 1962 (0.6%) ventricular arrhythmias. Their mean age was 55.8 (standard deviation [SD] 8.1) years, more than half (54.0%) were female, and the vast majority (96.6%) were white (Table 1). Compared to those without arrhythmias, participants who developed arrhythmias were more likely to be male and take aspirin, insulin, antihypertensive, and cholesterol-lowering medication; live in more deprived areas; have a higher number of long-term conditions; have less healthy lifestyles; higher waist circumferences and BMI; higher concentrations of total cholesterol and HbA1c; and have lower HDL cholesterol concentrations (Table 1 and Table S3). The majority of covariates had less than 3% of missing data (Table S4). The 10-year cumulative incidence was lower in the higher categories of serum mineral than in the lower categories (Table 2).

### 3.1. Calcium and Arrhythmias

**Figure 2:**
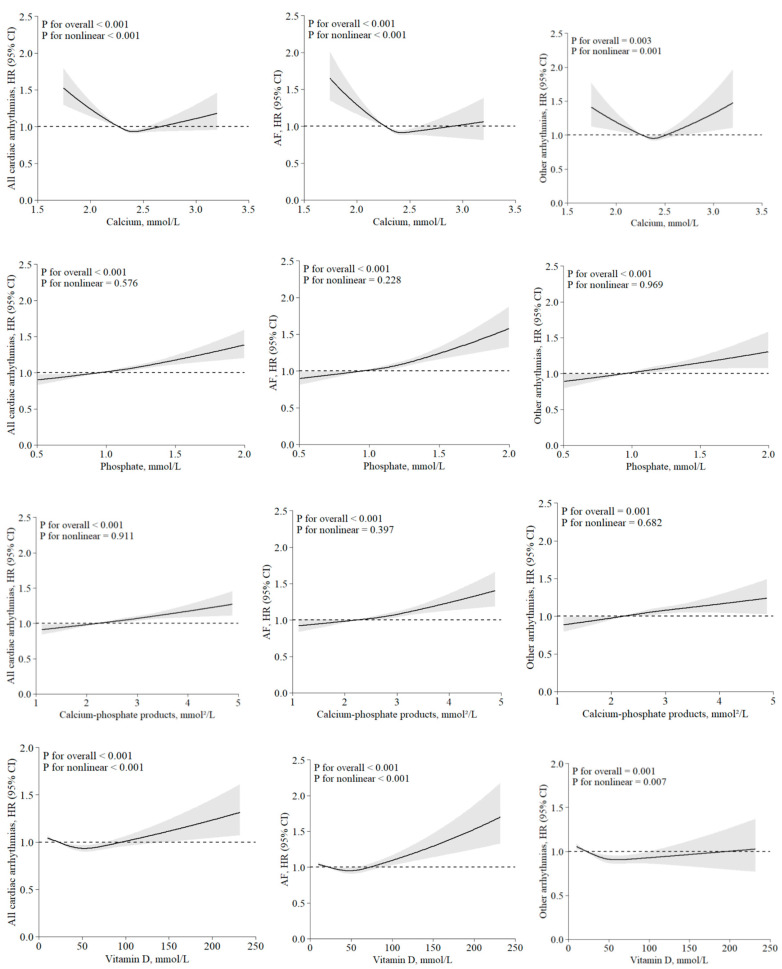
Dose–response association between serum calcium, phosphate, calcium–phosphate products, and vitamin D levels and incident arrhythmias. The model was adjusted for age, sex, Townsend deprivation index, ethnicity, smoking status, weekly units of alcohol use, physical activity, total sedentary time, sleep duration, fruit and vegetable intake, BMI, WC, HDL, total cholesterol, SBP, number of long-term conditions, and *C*-reactive protein at baseline. CI, confidence interval; HDL, high-density lipoprotein cholesterol; HR, hazard ratio; SBP, systolic blood pressure.

In the fully adjusted model (model 3), serum calcium in the highest quartile was associated with lower risk of all arrhythmias (HR 0.92, 95% CI 0.88–0.95) and AF (HR 0.89, 95% CI 0.85–0.93) compared with the lowest quartile; there was no association between serum calcium and other arrhythmias (HR 0.96, 95% CI 0.90–1.01), including bradyarrhythmia (HR 0.97, 95% CI 0.87–1.07) and ventricular arrhythmias (HR 0.96, 95% CI 0.81–1.13) (Table 3). The restricted cubic spline showed a nonlinear relationship between serum calcium and arrhythmias (*P*_nonlinear_ < 0.001; Figure 2). Similarly, nonlinear relationships were observed between serum calcium and other subtypes of arrhythmias (*P*_nonlinear_ < 0.001; Figure S1).

### 3.2. Phosphate and Arrhythmias

Higher quartiles of serum phosphate were associated with increased risk of all arrhythmias (HR and 95% CI for Q1 to Q4: reference; 1.03, 0.99–1.07; 1.05, 1.01–1.10; 1.11, 1.07–1.16), AF (HR and 95% CI for Q1 to Q4: reference; 1.04, 0.99–1.09; 1.07, 1.02–1.12; 1.16, 1.10–1.22), and other arrhythmias (HR and 95% CI for Q1 to Q4: reference; 1.02, 0.97–1.08; 1.06, 1.01–1.12; 1.08, 1.03–1.15) compared with the lowest quartile, after full adjustment (Table 3). The restricted cubic spline showed linear relations between serum phosphate and arrhythmias and its subtypes (*P*_nonlinear_ > 0.05; Figure 2 and Figure S1).

### 3.3. Calcium–Phosphate Product and Arrhythmias

In the fully adjusted model, the highest quartile of serum calcium–phosphate product was associated with higher risk of all arrhythmias (HR 1.09, 95% CI 1.04–1.13), AF (HR 1.11, 95% CI 1.05–1.16), and other arrhythmias (HR 1.08, 95% CI 1.02–1.15) compared with the lowest quartile (Table 3). Linear relationships were found for all arrhythmias, AF, other arrhythmias, and bradyarrhythmias (*P*_nonlinear_ > 0.05; Figure 2 and Figure S1).

### 3.4. Serum Vitamin D and Arrhythmias

In the fully adjusted models, compared to the lowest quartile, higher quartiles of vitamin D were associated with lower risk of all incident arrhythmias: Q2 HR 0.94 (95% CI 0.90–0.98), Q3 HR 0.95 (95% CI 0.91–0.99), and Q4 HR 0.96 (95% CI 0.92–1.00). Serum vitamin D was not associated with bradyarrhythmias (Q4 HR 0.93, 95% CI 0.83–1.04), but was associated with AF (Q4 HR 0.91, 95% CI 0.86–0.97) and ventricular arrhythmias (Q4 HR 0.68, 95% CI 0.57–0.81) (Table 3 and Table S5). We observed a nonlinear relationship between serum vitamin D concentrations and risk of arrhythmias (*P*_non-linerity_ < 0.001), with the lowest risk occurring at moderate levels of around 50 mmol/L. For AF, the risk decreased with increasing serum vitamin D up to around 75 mmol/L, and the risk increased thereafter. For the other arrhythmias, a nonlinear relationship was also observed, where the risk decreased up around 100 mmol/L and then plateaued.

### 3.5. Stratified Analyses and Sensitivity Analyses

**Figure 3:**
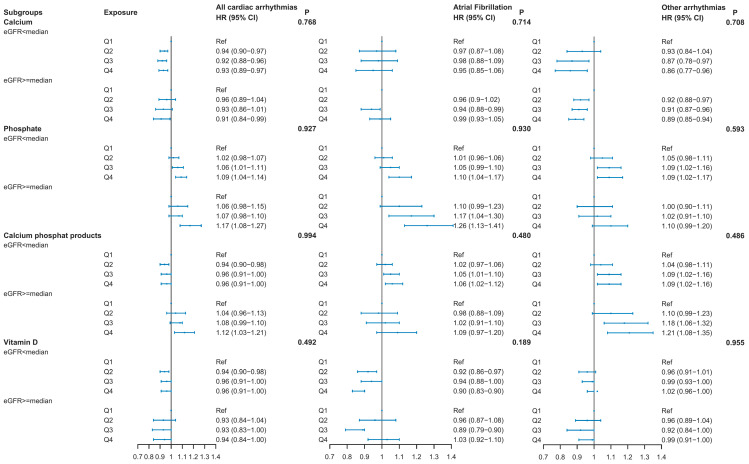
The hazard risk (HR) with 95% confidence intervals (95% CI) for incident cardiac arrhythmias in the subgroup analysis. Model adjusted for age, sex, Townsend deprivation index, ethnicity, smoking status, alcohol consumption, sleep duration, fruit and vegetable intake, processed meat intake, red meat intake, physical activity level, and total sedentary time, HDL-cholesterol concentration, total cholesterol concentration, SBP, BMI, WC, HbA1c, antihypertensive medication use, cholesterol-lowering medication, aspirin, insulin, and number of long-term conditions. Ref, reference group; eGFR, estimated glomerular filtration rate.

We did not identify any significant interactions with kidney function (Figure 3). In the subgroup analysis, the associations between serum calcium, phosphate, and calcium–phosphate products and arrhythmias were mostly consistent. A significantly negative association was observed between serum vitamin D and all cardiac arrhythmias among those with eGFR < median value, but not among those with eGFR ≥ median value. Both groups stratified by eGFR value showed no association between serum vitamin D and other arrhythmias.

In our sensitivity analyses, the associations between serum calcium, phosphorus, calcium–phosphate product, and vitamin D and arrhythmias did not change appreciably when we excluded participants with incident arrhythmias in the first two years (Table S6). The results were also consistent when performing the regression models after using multiple imputation of missing covariate data (Table S6). After applying the Bonferroni correction, most of the associations remained statistically significant, except for vitamin D and all cardiac arrhythmias and AF, and phosphate and other arrhythmias.

## 4. Discussion

In this large-scale population cohort, we observed significant associations between serum calcium, phosphorus, calcium–phosphate product, and vitamin D and incident arrhythmias overall, and AF and other arrhythmias specifically. The relationships with arrhythmias were nonlinear for serum calcium and vitamin D and linear for phosphorus and calcium–phosphate product.

Previous studies have observed associations between serum calcium, phosphorus, and vitamin D and both cardiovascular risk factors [23,24,25] and CVD [2,26,27,28]. Evidence has accumulated that serum calcium, phosphorus, and vitamin D concentrations may also be associated with AF [13,29,30,31,32], the main subtype of arrhythmias; however, the findings remain controversial, and the relationships with other arrhythmias are still underexplored. Our finding of an association between serum phosphorus concentrations and arrhythmias is consistent with some previous cohort studies reporting an association with AF, such as the Atherosclerosis Risk in Communities (ARIC) study that included 14,675 participants [13]. However, inconsistent findings have been observed for serum calcium and vitamin D, with non-significant associations reported by the ARIC study [13,29], the Rotterdam Study [30], and the Framingham Heart Study [31]. The inconsistent findings may be because of lower statistical power (e.g., 2930 participants in the FHS study; 3395 participants in the Rotterdam Study) or failure to take account of nonlinear relationships in previous studies [13,29,30,31,32]. Whilst a previous Mendelian randomization (MR) study did not find an association between genetically predicted serum calcium, phosphorus, and vitamin D concentrations and AF, this may be due to the limited number of instrumental variables (7 SNPs) or pleiotropy, and MR analysis cannot take account of nonlinear relationships since the exposure is based on summary-level data [5,28,33]. Meanwhile, our findings have provided new evidence of the dose–response relationships. The nonlinear relationships between serum calcium and vitamin D and arrhythmias may explain the non-significant findings of some previous studies [13,29,30,31]. To our knowledge, the present study is the first to provide new evidence of the association between serum calcium, vitamin D, phosphorus, and calcium–phosphate product levels and other arrhythmias and their dose–response pattern.

Kidney function is linked to the metabolism of serum calcium, phosphorus, and vitamin D [34] and can also increase the risk of arrhythmias [35], so it was critical to understand whether kidney function affects the associations between serum minerals and arrhythmias. The present study did not find significant effect modification by kidney function on any of the associations investigated. The finding on serum phosphorus is not consistent with an earlier study, which suggested that the association between serum phosphorus and AF was only significant in those with an eGFR of ≥90 mL/min/1.72 m^2^ [13]. Previous studies did not find any association between vitamin D and AF, no matter whether having decreased kidney function [30]. Therefore, patients with decreased kidney function or chronic kidney disease may not be a high-risk group to be susceptible to the effect of serum calcium, phosphorus, vitamin D, and the risk of arrhythmias.

The underlying mechanism between serum minerals and arrhythmias is still not well understood. The mechanisms of serum calcium and arrhythmias may involve the heart muscle function due to affecting the QT interval [36]. Increased QT interval and QT dispersion were the most common findings associated with vitamin D deficiency [37]. Vitamin D regulates calcium and phosphorus homeostasis, so deficiency or excess can indirectly alter cardiac electrophysiology [38]. Elevated serum phosphorus may lead to calcium–phosphorus imbalance and promote vascular and valvular calcification and myocardial remodelling [39], all of which contribute to atrial and ventricular arrhythmias.

The strengths of this study include its large sample size and long follow-up period and the ability to adjust for a range of potential confounders and to test for interactions with kidney function. This study is the first to investigate the associations between four mineral exposures and a range of arrhythmia outcomes, in contrast to previous studies that focused solely on AF. Moreover, our study explored dose–relationships and performed a series of sensitivity analyses. The findings in the present study add to the existing evidence on mineral metabolism and risk of incident arrhythmias.

However, potential limitations should be noted. First, dynamic changes in serum calcium, phosphorus, and vitamin D cannot be tested, because these biomarkers were measured only once in the UK Biobank. This aspect warrants further investigation in future studies. The analyses were based on a single baseline measure, which does not account for within-person variability over time and may be prone to regression-to-the-mean effects. Second, the UK Biobank is not representative of the general population in that participants have generally healthier lifestyles; however, the exposure-outcome associations are generalizable [40]. Third, UK Biobank participants were aged 40–69 years of age and predominantly of White British origin, limiting the generalizability of the findings to other ethnic groups, countries, and age groups. Fourth, while we cannot exclude the possibility of residual confounding from unmeasured variables such as parathyroid hormone and serum magnesium, sodium, and potassium, our findings were corroborated in a landmark analysis. Forth, some confounders such as lifestyle factors were self-reported, which may result in inaccurate or imprecise measurement. Moreover, incidental cardiac arrhythmias were ascertained using ICD-10 codes from hospital admissions and death registry records. However, it may not capture milder, subclinical, or transient arrhythmias that are diagnosed only in primary care settings or managed exclusively in outpatient clinics. So, our findings may primarily reflect more clinically significant arrhythmias that require hospital-level care.

## 5. Conclusions

Serum calcium, vitamin D, phosphate, and calcium–phosphate product were associated with an increased risk of incident arrhythmias. We observed J- or U-shaped relationships for serum calcium with the lowest risk at around 2.4 mmol/L and vitamin D with the lowest risk at around 50 mmol/L and linear dose–relationships for phosphorus and calcium–phosphate products. These novel findings advance current knowledge by highlighting associations with arrhythmias beyond AF and evidence of optimal concentrations of serum calcium, phosphate, and vitamin D to minimize arrhythmic risk.

## Figures and Tables

**Table 1:** Baseline characteristics of participants in the UK Biobank.

	Overall	Cardiac Arrhythmias		
		No	Yes	*p*
N	348,094	315,420	32,674	
Age (years), mean (SD)	55.8 (8.1)	55.3 (8.1)	60.8 (6.7)	<0.001
Men, *n* (%)	160,190 (46.0)	140,597 (44.6)	19,593 (60.0)	<0.001
Deprivation index, *n* (%)				0.116
low	117,922 (33.9)	107,020 (34.0)	10,902 (33.4)	
moderate	116,575 (33.5)	105,558 (33.5)	11,017 (33.8)	
high	113,164 (32.6)	102,445 (32.5)	10,719 (32.8)	
Ethnicity, *n* (%)				<0.001
White	328,136 (96.8)	296,661 (96.6)	31,475 (98.0)	
South Asia	5092 (1.5)	4757 (1.5)	335 (1.0)	
Other	5850 (1.7)	5557 (1.8)	293 (0.9)	
Vitamin D (nmol/L), mean (SD)	48.55 (21.02)	48.47 (21.02)	49.23 (21.06)	<0.001
Calcium (mmol/L), mean (SD)	2.38 (0.09)	2.38 (0.09)	2.38 (0.10)	<0.001
Phosphate (nmol/L), mean (SD)	1.16 (0.16)	1.16 (0.16)	1.15 (0.16)	<0.001
Calcium phosphate product (mmol^2^/L^2^), mean (SD)	2.76 (0.42)	2.76 (0.42)	2.73 (0.42)	<0.001

**Table 2:** The 10-year cumulative incidence of arrhythmias by different serum mineral groups.

Serum Mineral Levels	All Cardiac Arrhythmias (%)	Atrial Fibrillation (%)	Other Arrhythmias (%)
Calcium (mmol/L)			
Q1 (1.19~2.32)	6.16 (6.00–6.32)	4.08 (3.95–4.21)	2.96 (2.85–3.08)
Q2 (2.32~2.37)	5.58 (5.43–5.74)	3.60 (3.48–3.73)	2.80 (2.69–2.91)
Q3 (2.37~2.43)	5.71 (5.55–5.86)	3.61 (3.49–3.74)	2.91 (2.79–3.02)
Q4 (2.43~3.61)	5.57 (5.42–5.73)	3.54 (3.42–3.67)	2.84 (2.73–2.96)
Phosphate (mmol/L)			
Q1 (0.43~1.05)	6.04 (5.88–6.2)	3.96 (3.83–4.09)	2.96 (2.84–3.07)
Q2 (1.05~1.16)	5.86 (5.70–6.01)	3.78 (3.65–3.90)	2.92 (2.81–3.03)
Q3 (1.16~1.27)	5.60 (5.45–5.75)	3.57 (3.44–3.69)	2.83 (2.72–2.94)
Q4 (1.27~4.70)	5.53 (5.37–5.68)	3.53 (3.41–3.66)	2.80 (2.69–2.91)
Calcium–phosphate products (mmol^2^/L^2^)			
Q1 (0.96~2.48)	5.99 (5.84–6.15)	3.97 (3.84–4.1)	2.91 (2.79–3.02)
Q2 (2.48~2.75)	5.92 (5.76–6.07)	3.81 (3.68–3.93)	2.95 (2.84–3.07)
Q3 (2.75~3.03)	5.68 (5.53–5.84)	3.60 (3.48–3.73)	2.87 (2.76–2.98)
Q4 (3.03~9.33)	5.44 (5.29–5.59)	3.46 (3.34–3.59)	2.78 (2.67–2.89)
Vitamin D (nmol/L)			
Q1 (10.0~32.4)	5.47 (5.32–5.62)	3.45 (3.33–3.57)	2.83 (2.72–2.95)
Q2 (32.4~46.8)	5.74 (5.58–5.89)	3.62 (3.50–3.75)	2.91 (2.80–3.03)
Q3 (46.8~62.3)	5.93 (5.78–6.09)	3.84 (3.71–3.97)	2.95 (2.84–3.07)
Q4 (62.3~340.0)	5.89 (5.73–6.05)	3.93 (3.80–4.06)	2.81 (2.70–2.92)

**Table 3:** Association of serum calcium, phosphate, calcium–phosphate products, and vitamin D levels and incident arrhythmias.

Serum Mineral Levels	No. of Cases/Person-Years	Model 1	*p*-Value	Model 2	*p*-Value	Model 3	*p*-Value
		HR (95% CI)		HR (95% CI)		HR (95% CI)	
**All cardiac arrhythmias**							
Calcium (mmol/L)							
Q1 (1.19~2.32)	8753/88,031	Ref.		Ref.		Ref.	
Q2 (2.32~2.37)	8064/86,389	0.94 (0.91–0.97)	<0.001	0.93 (0.90–0.96)	<0.001	0.94 (0.90–0.97)	<0.001
Q3 (2.37~2.43)	8048/87,271	0.95 (0.92–0.98)	<0.001	0.92 (0.89–0.96)	<0.001	0.92 (0.89–0.96)	<0.001
Q4 (2.43~3.61)	7809/86,402	0.95 (0.92–0.98)	<0.001	0.94 (0.91–0.97)	0.001	0.92 (0.88–0.95)	<0.001
Phosphate (mmol/L)							
Q1 (0.43~1.05)	8805/87,250	Ref.		Ref.		Ref.	
Q2 (1.05~1.16)	8276/87,046	1.01 (0.97–1.04)	0.640	1 (0.97–1.04)	0.790	1.03 (0.99–1.07)	0.100
Q3 (1.16~1.27)	7903/87,213	1.02 (0.99–1.05)	0.170	1.03 (0.98–1.06)	0.160	1.05 (1.01–1.10)	0.008
Q4 (1.27~4.70)	7690/86,584	1.08 (1.05–1.12)	<0.001	1.07 (1.03–1.11)	<0.001	1.11 (1.07–1.16)	<0.001
Calcium–phosphate products (mmol^2^/L^2^)							
Q1 (0.96~2.48)	8764/87,023	Ref.		Ref.		Ref.	
Q2 (2.48~2.75)	8287/87,024	1.01 (0.98–1.04)	0.530	1.01 (0.97–1.04)	0.640	1.03 (0.99–1.07)	0.100
Q3 (2.75~3.03)	7994/87,025	1.03 (1.00–1.06)	0.053	1.03 (0.99–1.07)	0.130	1.06 (1.02–1.10)	0.006
Q4 (3.03~9.33)	7629/87,021	1.06 (1.03–1.10)	<0.001	1.05 (1.01–1.09)	0.009	1.09 (1.04–1.13)	<0.001
Vitamin D (nmol/L)							
Q1 (10.0~32.4)	7808/87,455	Ref.		Ref.		Ref.	
Q2 (32.4~46.8)	8118/86,955	0.93 (0.89–0.96)	<0.001	0.94 (0.90–0.98)	<0.001	0.94 (0.90–0.98)	0.004
Q3 (46.8~62.3)	8322/86,877	0.89 (0.86–0.92)	<0.001	0.92 (0.88–0.95)	<0.001	0.95 (0.91–0.99)	0.018
Q4 (62.3~340.0)	8418/86,721	0.88 (0.85–0.91)	<0.001	0.91 (0.87–0.94)	<0.001	0.96 (0.92–1.00)	0.045
**Atrial Fibrillation**							
Calcium (mmol/L)							
Q1 (1.19~2.32)	5884/88,031	Ref.		Ref.		Ref.	
Q2 (2.32~2.37)	5226/86,389	0.91 (0.87–0.95)	<0.001	0.91 (0.87–0.95)	<0.001	0.92 (0.88–0.96)	<0.001
Q3 (2.37~2.43)	5139/87,271	0.9 (0.86–0.94)	<0.001	0.89 (0.86–0.94)	<0.001	0.90 (0.86–0.94)	<0.001
Q4 (2.43~3.61)	4981/86,402	0.91 (0.87–0.95)	<0.001	0.90 (0.87–0.95)	<0.001	0.89 (0.85–0.93)	<0.001
Phosphate (mmol/L)							
Q1 (0.43~1.05)	5789/87,250	Ref.		Ref.		Ref.	
Q2 (1.05~1.16)	5340/87,046	1 (0.95–1.03)	0.780	0.99 (0.95–1.04)	0.780	1.04 (0.99–1.09)	0.120
Q3 (1.16~1.27)	5160/87,213	1.03 (0.99–1.07)	0.150	1.03 (0.98–1.07)	0.270	1.07 (1.02–1.12)	0.006
Q4 (1.27~4.70)	4941/86,584	1.09 (1.04–1.13)	<0.001	1.09 (1.04–1.14)	<0.001	1.16 (1.10–1.22)	<0.001
Calcium–phosphate products (mmol^2^/L^2^)							
Q1 (0.96~2.48)	5789/87,023	Ref.		Ref.		Ref.	
Q2 (2.48~2.75)	5374/87,024	1.00 (0.95–1.03)	0.790	0.99 (0.95–1.04)	0.740	1.03 (0.97–1.08)	0.270
Q3 (2.75~3.03)	5191/87,025	1.03 (0.98–1.07)	0.190	1.02 (0.97–1.07)	0.310	1.07 (1.02–1.13)	0.005
Q4 (3.03~9.33)	4876/87,021	1.06 (1.01–1.10)	0.008	1.05 (1.00–1.10)	0.042	1.11 (1.05–1.16)	<0.001
Vitamin D (nmol/L)							
Q1 (10.0~32.4)	4990/87,455	Ref.		Ref.		Ref.	
Q2 (32.4~46.8)	5217/86,955	0.91 (0.87–0.95)	<0.001	0.93 (0.88–0.98)	0.003	0.94 (0.89–0.99)	0.023
Q3 (46.8~62.3)	5396/86,877	0.87 (0.83–0.91)	<0.001	0.90 (0.86–0.95)	<0.001	0.96 (0.91–1.01)	0.110
Q4 (62.3~340.0)	5620/86,721	0.88 (0.85–0.92)	<0.001	0.92 (0.88–0.97)	<0.001	1.00 (0.95–1.05)	0.990
**Other arrhythmias**							
Calcium (mmol/L)							
Q1 (1.19~2.32)	4428/88,031	Ref.		Ref.		Ref.	
Q2 (2.32~2.37)	4215/86,389	0.98 (0.93–1.02)	0.240	0.96 (0.90–1.01)	0.048	0.96 (0.91–1.01)	0.100
Q3 (2.37~2.43)	4264/87,271	1.00 (0.96–1.04)	0.890	0.95 (0.92–1.00)	0.230	0.95 (0.90–1.00)	0.039
Q4 (2.43~3.61)	4163/86,402	1.00 (0.96–1.05)	0.880	1.00 (0.90–1.01)	0.840	0.96 (0.90–1.01)	0.100
Phosphate (mmol/L)							
Q1 (0.43~1.05)	4532/87,250	Ref.		Ref.		Ref.	
Q2 (1.05~1.16)	4333/87,046	1.03 (0.98–1.07)	0.230	1.02 (0.96–1.07)	0.480	1.02 (0.97–1.08)	0.380
Q3 (1.16~1.27)	4140/87,213	1.04 (1.00–1.09)	0.050	1.06 (1.01–1.11)	0.028	1.06 (1.01–1.12)	0.028
Q4 (1.27~4.70)	4065/86,584	1.12 (1.07–1.17)	<0.001	1.08 (1.02–1.13)	0.005	1.08 (1.03–1.15)	0.005
Calcium–phosphate products (mmol^2^/L^2^)							
Q1 (0.96~2.48)	4490/87,023	Ref.		Ref.		Ref.	
Q2 (2.48~2.75)	4316/87,024	1.03 (0.98–1.08)	0.160	1.03 (0.97–1.08)	0.300	1.03 (0.98–1.09)	0.210
Q3 (2.75~3.03)	4207/87,025	1.07 (1.02–1.11)	0.004	1.07 (1.02–1.12)	0.010	1.06 (1.01–1.12)	0.023
Q4 (3.03~9.33)	4057/87,021	1.11 (1.06–1.16)	<0.001	1.08 (1.03–1.14)	0.002	1.08 (1.02–1.15)	0.006
Vitamin D (nmol/L)							
Q1 (10.0~32.4)	4160/87,455	Ref.		Ref.		Ref.	
Q2 (32.4~46.8)	4288/86,955	0.94 (0.90–0.98)	0.007	0.96 (0.90–1.01)	0.088	0.95 (0.89–1.00)	0.057
Q3 (46.8~62.3)	4347/86,877	0.9 (0.86–0.94)	<0.001	0.93 (0.88–0.98)	0.010	0.95 (0.89–1.01)	0.075
Q4 (62.3~340.0)	4271/86,721	0.87 (0.83–0.91)	<0.001	0.89 (0.84–0.94)	<0.001	0.91 (0.86–0.97)	0.001

Abbreviations: Ref, reference group; CI, confidence interval; HbA1c, glycated hemoglobin (Hemoglobin A1c); HR, hazard ratio. Model 1: adjusted for age, sex, Townsend deprivation index, and ethnicity; Model 2: additionally adjusted for smoking status, alcohol consumption, sleep duration, fruit and vegetable intake, processed meat intake, red meat intake, physical activity level, and total sedentary time; Model 3: additionally adjusted for HDL-cholesterol concentration, total cholesterol concentration, SBP, BMI, WC, HbA1c, eGFR, antihypertensive medication use, cholesterol-lowering medication, aspirin, insulin, and number of long-term conditions.

## Data Availability

Data analyzed in this study were from the UK Biobank and are available upon application to UK Biobank: https://www.ukbiobank.ac.uk.

## Supplementary Materials

The following supporting information can be downloaded at: https://www.mdpi.com/article/10.3390/nu17243895/s1, Table S1: ICD-10 of outcomes in this study; Table S2: List of self-reported long-term condition for multimorbidity count; Table S3: Baseline characteristics of participants in the UK Biobank; Table S4: Percentage of covariates with missing data that were included in the association analysis; Table S5: Association of serum calcium, phosphate, calcium–phosphate products, and vitamin D levels and incident bradyarrhythmias and ventricular arrhythmias; Table S6: Sensitivity analysis of serum calcium, phosphate, calcium–phosphate products, and vitamin D levels and incident arrhythmias; Figure S1: Dose-response association between serum calcium, phosphate, calcium–phosphate products, and vitamin D levels and bradyarrhythmia and ventricular arrhythmias.
