# Supplementary material for: Serum Levels of Calcium, Phosphate, and Vitamin D and Incident Arrhythmias: A Prospective Cohort Study of 348,094 UK Biobank Participants
Source: Nutrients. 2025 Dec 12;17(24):3895. doi: 10.3390/nu17243895 (PMC12736382; doi:10.3390/nu17243895)
Supplement: Supplementary file 1 [file nutrients-17-03895-s001.zip › nutrients-3972248-supplementary.pdf]

Supplemental materials

Table S1. ICD-10 of outcomes in this study

Disease	Field Name	Code
Cardiac arrhythmias	Diagnoses - ICD10	I44-I49
Atrial fibrillation	Diagnoses - ICD10	I48
Other cardiac arrhythmias	Diagnoses - ICD10	I44-I47, I49
Bradyarrhythmias	Diagnoses - ICD10	I44.0, I44.1, I44.2, I44.3, I44.5, I49.5
Ventricular arrhythmias	Diagnoses - ICD10	I47.0, I47.2, I49.0, I46.0, I46.1, I46.9

Table S2. List of self-reported long-term condition for multimorbidity count

Long term condition grouping	Conditions included as reported by participants
**1. Painful conditions**	Back pain
	Joint pain
	Back pain
	Joint pain
	Headaches (not migraine)
	Sciatica
	Plantar fasciitis
	Carpal tunnel syndrome
	Fibromyalgia
	Arthritis
	Shingles
	Disc problem
	Prolapsed disc/slipped disc
	Spine arthritis/spondylitis
	Ankylosing spondylitis
	Back problem
	Osteoarthritis
	Gout
	Cervical spondylosis
	Trigeminal neuralgia
	Disc degeneration
	Trapped nerve/compressed nerve
**2. Hypertension**	Hypertension
	Essential Hypertension
**3. Depression**	Depression
	Postnatal Depression
**4. Asthma**	Asthma
**5. Atrial Fibrillation**	Atrial Fibrillation
**6. Coronary Heart Disease**	Heart attack/Myocardial Infarction
	Angina
**7. Dyspepsia**	Gastro-oesophageal reflux (GORD)/gastric reflux
	Oesophagitis /Barrett's oesophagus
	Gastric stomach ulcers
	Gastric erosions/gastritis
	Duodenal ulcer
	Dyspepsia/indigestion
	Hiatus hernia
	Helicobacter pylori
**8. Diabetes**	Diabetic nephropathy
	Diabetic neuropathy/ulcers
	Diabetes
	Type 1 diabetes
	Type 2 diabetes
	Diabetic eye disease
	Thyroid problem (not cancer)
**9. Thyroid disorders**	Hyperthyroidism/thyrotoxicosis
	Hypothyroidism/myxoedema
	Grave's disease
	Thyroid goitre
	Thyroiditis
**10. Connective tissue disorders**	Myositis/myopathy
	Systemic Lupus Erythematosus
	Connective tissue disorder
	Sjogrens syndrome/sicca syndrome
	Dermatopolymyositis
	Scleroderma/systemic sclerosis
	Rheumatoid arthritis

	Psoriatic arthropathy
	Dermatomyositis
	Polymyositis
	Polymyalgia Rheumatica
	Malabsorption/coeliac disease
**11. Chronic Obstructive Pulmonary Disease (COPD)**	COPD/chronic obstructive airways disease
	Emphysema/chronic bronchitis
	Emphysema
**12. Anxiety**	Anxiety/panic attacks
	Nervous breakdown
	Post-traumatic stress disorder
	Obsessive compulsive disorder
	Stress
	Insomnia
	Psychological/psychiatric problem
**13. Irritable bowel syndrome**	Irritable bowel syndrome
**14. Alcohol problems**	Alcohol dependency
	Alcoholic liver disease/alcoholic cirrhosis
**15. Other psychoactive substance abuse**	Opioid dependency
	Other substance abuse/dependency
**16. Treated constipation**	Constipation
**17. Stroke/Transient Ischaemic Attack (TIA)**	Stroke
	TIA
	Subarachnoid haemorrhage
	Brain haemorrhage
	Ischaemic stroke
**18. Chronic kidney disease**	Polycystic kidney
	Diabetic nephropathy
	Renal/kidney failure
	Renal failure requiring dialysis
	Renal failure not requiring dialysis
	Kidney nephropathy
	Immunoglobulin A (IgA) nephropathy
**19. Diverticular disease**	Diverticular disease
	Diverticulitis
**20. Peripheral vascular disease**	Peripheral vascular disease
	Leg claudication/intermittent claudication
**21. Heart failure**	Cardiomyopathy
	Hypertrophic cardiomyopathy
	Heart failure/pulmonary oedema
**22. Prostate disorders**	Prostate problem (not cancer)
	Enlarged prostate
	Benign prostatic hypertrophy
**23. Glaucoma**	Glaucoma
**24. Epilepsy**	Epilepsy
**25. Dementia**	Dementia
	Alzheimer's disease
	Cognitive impairment
**26. Schizophrenia/bipolar disorder**	Schizophrenia
	Mania/
	Bipolar disorder
	Manic depression
**27. Psoriasis/eczema**	Eczema
	Dermatitis
	Psoriasis
**28. Inflammatory Bowel Disease**	Inflammatory Bowel Disease
	Crohn's disease
	Ulcerative colitis
**29. Migraine**	Migraine
**30. Chronic sinusitis**	Chronic sinusitis

**31. Anorexia or bulimia**	Anorexia
	Bulimia
	Other eating disorders
**32. Bronchiectasis**	Bronchiectasis
**33. Parkinson's disease**	Parkinson's disease
**34. Multiple Sclerosis**	Multiple Sclerosis
**35. Viral Hepatitis**	Infective/viral hepatitis
	Hepatitis B
	Hepatitis C
	Hepatitis D
	Hepatitis E
**36. Chronic Liver disease**	Oesophageal varices
	Non infective hepatitis
	Liver failure/cirrhosis
	Primary biliary cirrhosis
**37. Osteoporosis**	Osteoporosis
**38. Chronic fatigue syndrome**	Chronic fatigue syndrome
**39. Endometriosis**	Endometriosis
**40. Meniere's disease**	Meniere's disease
**41. Pernicious Anaemia**	Pernicious Anaemia
**42. Polycystic ovary**	Polycystic ovary
**43. Cancer**	Lifetime diagnosis

Table S3 Baseline characteristics of participants in the UK Biobank

	Overall	Cardiac arrhythmias		p
		No	Yes	
N	348094	315420	32674	
Smoking status, n (%)				<0.001
Never	194939 (56.2)	179180 (57.0)	15759 (48.5)	
Previous	115772 (33.4)	102650 (32.7)	13122 (40.3)	
Current	36186 (10.4)	32546 (10.4)	3640 (11.20)	
Alcohol (weekly units), median (IQR)	10.5 (3.0-22.5)	10.5 (3.00-22.35)	12.0 (2.70-27.00)	<0.001
Physical activity, n (%)				<0.001
low	52168 (18.4)	47203 (18.4)	4965 (19.0)	
moderate	115220 (40.7)	104903 (40.8)	10317 (39.6)	
high	115853 (40.9)	105063 (40.9)	10790 (41.4)	
Sedentary time (hours/day), mean (SD)	4.48 (2.58)	4.45 (2.58)	4.78 (2.60)	<0.001
Fruit and vegetable intake (portion per day), mean (SD)	4.10 (2.42)	4.09 (2.42)	4.14 (2.50)	0.001
Red meat intake (times/week), n (%)				<0.001
0	32333 (9.3)	29995 (9.5)	2338 (7.2)	
0-2	206702 (59.5)	187834 (59.7)	18868 (57.9)	
>2	108311 (31.20)	96908 (30.8)	11403 (35.0)	
Processed meat intake (times/week), n (%)				<0.001
0	23718 (6.9)	22040 (7.1)	1678 (5.2)	
0-2	211950 (61.6)	192816 (61.8)	19134 (59.4)	
>2	108487 (31.5)	97064 (31.10)	11423 (35.4)	
Sleep duration, n (%)				<0.001
1-6 hours	84529 (24.4)	76273 (24.3)	8256 (25.5)	
7-8 hours	237230 (68.5)	216010 (68.9)	21220 (65.4)	
⩾9 hours	24401 (7.0)	21447 (6.8)	2954 (9.1)	
BMI (kg/m^2), mean (SD)	27.29 (4.74)	27.16 (4.66)	28.55 (5.24)	<0.001
WC (cm), mean (SD)	89.85 (13.34)	89.29 (13.13)	95.22 (14.18)	<0.001
HDL (mmol/L), mean (SD)	1.45 (0.38)	1.46 (0.38)	1.40 (0.37)	<0.001
Total Cholesterol (mmol/L), mean (SD)	5.65 (0.99)	5.66 (0.98)	5.53 (1.03)	<0.001
HbA1c (mmol/mol), mean (SD)	35.77 (6.38)	35.61 (6.18)	37.25 (7.95)	<0.001
SBP (mmHg), mean (SD)	137.43 (18.46)	136.80 (18.30)	143.49 (18.83)	<0.001
DBP (mmHg), mean (SD)	82.31 (10.07)	82.19 (10.04)	83.44 (10.22)	<0.001
eGFR (mL/min/1.73 m^2), mean (SD)	84.67 (4.98)	84.98 (4.96)	81.68 (4.16)	<0.001
Number of long-term conditions, n (%)				<0.001
0	139099 (40.0)	130559 (41.4)	8540 (26.1)	
1	117506 (33.8)	106286 (33.7)	11220 (34.3)	
≥2	91489 (26.3)	78575 (24.9)	12914 (39.5)	
History of diabetes, n (%)	14998 (4.3)	12209 (3.9)	2789 (8.5)	<0.001
History of hypertension, n (%)	84811 (24.4)	71554 (22.7)	13257 (40.6)	<0.001

History of hyperlipidemia, n (%)	36746 (10.6)	31203 (9.9)	5543 (17.0)	<0.001
Aspirin, n (%)	34447 (9.9)	28440 (9.0)	6007 (18.4)	<0.001
Antihypertensive medication use, n (%)	34767 (10.0)	29168 (9.2)	5599 (17.1)	<0.001
Cholesterol-lowering medicatio, n (%)	46674 (13.4)	38901 (12.3)	7773 (23.8)	<0.001
Insulin treatment, n (%)	544 (0.2)	475 (0.2)	69 (0.2)	0.01
Vitamin D assay season, n (%)				0.602
Spring	105378 (30.3)	95466 (30.3)	9912 (30.3)	
Summer	109905 (31.6)	99510 (31.6)	10395 (31.8)	
Autumn	59332 (17.1)	53780 (17.1)	5552 (17.0)	
Winter	73333 (21.10)	66532 (21.10)	6801 (20.8)	
Calcium supplementation, n (%)	6036 (1.7)	7066 (2.2)	693 (2.1)	0.175
Vitamin D supplementation, n (%)	12290 (3.5)	5463 (1.7)	573 (1.8)	0.752
Vitamin D (mmol/L), mean (SD)	48.55 (21.02)	48.47 (21.02)	49.23 (21.06)	<0.001
Calcium (mmol/L), mean (SD)	2.38 (0.09)	2.38 (0.09)	2.38 (0.10)	<0.001
Phosphate (nmol/L), mean (SD)	1.16 (0.16)	1.16 (0.16)	1.15 (0.16)	<0.001
Calcium phosphate product ($mmol^2/L^2$), mean (SD)	2.76 (0.42)	2.76 (0.42)	2.73 (0.42)	<0.001

BMI, body mass index; DBP, diastolic blood pressure; eGFR, estimated glomerular filtration rate; HDL, high-density lipoprotein; SBP, systolic blood pressure; WC, waist circumference.

Table S4 Percentage of covariates with missing data that were included in the association analysis

variable	Number of missing data	Percent of missing data (%)
Deprivation index	433	0.12
Ethnicity	9016	2.59
Smoking status	1197	0.34
Alcohol (weekly units)	30329	8.71
Physical activity	64853	18.63
Sleep duration	1934	0.56
Red meat intake	3939	1.13
Processed meat intake	748	0.21
BMI	1081	0.31
WC	696	0.20
SBP	29713	8.54
HDL	266	0.08
Total Cholesterol	99	0.03
CRP	739	0.21
eGFR	124	0.36

BMI, body mass index; DBP, diastolic blood pressure; eGFR, estimated glomerular filtration rate; HDL, high-density lipoprotein; SBP, systolic blood pressure; WC, waist circumference.

Table S5 Association of serum calcium, phosphate, calcium–phosphate products, and vitamin D levels and incident bradyarrhythmias and ventricular arrhythmias

Serum mineral levels	No. of cases/subjects	Model 1	P value	Model 2	P value	Model 3	P value
		HR (95% CI)		HR (95% CI)		HR (95% CI)	
**Bradyarrhythmias**							
Calcium (mmol/L)							
Q1 (1.19~2.32)	1342/88031	Ref.		Ref.		Ref.	
Q2 (2.32~2.37)	1227/86389	0.95 (0.88-1.03)	0.240	0.95 (0.86-1.04)	0.630	0.96 (0.87-1.06)	0.680
Q3 (2.37~2.43)	1275/87271	1.02 (0.94-1.10)	0.670	1.00 (0.92-1.10)	0.140	0.98 (0.89-1.08)	0.019
Q4 (2.43~3.61)	1163/86402	0.99 (0.91-1.07)	0.730	1.01 (0.92-1.11)	0.350	0.97 (0.87-1.07)	0.620
Phosphate (mmol/L)							
Q1 (0.43~1.05)	1417/87250	Ref.		Ref.		Ref.	
Q2 (1.05~1.16)	1277/87046	1.01 (0.93-1.09)	0.890	1.03 (0.93-1.12)	0.590	1.04 (0.94-1.15)	0.400
Q3 (1.16~1.27)	1198/87213	1.05 (0.96-1.13)	0.250	1.07 (0.97-1.18)	0.140	1.07 (0.96-1.18)	0.210
Q4 (1.27~4.70)	1115/86584	1.10 (1.01-1.19)	0.023	1.10 (1.00-1.21)	0.048	1.11 (1.00-1.23)	0.041
Calcium–phosphate products ($mmol^2/L^2$)							
Q1 (0.96~2.48)	1391/87023	Ref.		Ref.		Ref.	
Q2 (2.48~2.75)	1293/87024	1.04 (0.95-1.12)	0.370	1.07 (0.98-1.17)	0.130	1.08 (0.98-1.19)	0.100
Q3 (2.75~3.03)	1206/87025	1.07 (0.98-1.15)	0.110	1.11 (1.01-1.22)	0.025	1.11 (1.00-1.22)	0.046
Q4 (3.03~9.33)	1117/87021	1.12 (1.03-1.22)	0.007	1.14 (1.03-1.25)	0.009	1.14 (1.02-1.26)	0.015
Vitamin D (nmol/L)							
Q1 (10.0~32.4)	1190/87455	Ref.		Ref.		Ref.	
Q2 (32.4~46.8)	1223/86955	0.91 (0.83-0.99)	0.021	0.92 (0.83-1.01)	0.094	0.93 (0.84-1.04)	0.190
Q3 (46.8~62.3)	1293/86877	0.88 (0.81-0.96)	0.003	0.91 (0.82-1.00)	0.049	0.95 (0.85-1.05)	0.290
Q4 (62.3~340.0)	1300/86721	0.87 (0.79-0.94)	<0.001	0.88 (0.79-0.97)	0.008	0.93 (0.83-1.04)	0.190
**Ventricular arrhythmias**							
Calcium (mmol/L)							
Q1 (1.19~2.32)	500/88031	Ref.		Ref.		Ref.	
Q2 (2.32~2.37)	525/86389	1.10 (0.97-1.25)	0.130	1.04 (0.90-1.20)	0.420	1.03 (0.88-1.21)	0.240
Q3 (2.37~2.43)	454/87271	0.98 (0.86-1.11)	0.710	0.89 (0.76-1.04)	0.700	0.82 (0.69-0.97)	0.940
Q4 (2.43~3.61)	483/86402	1.08 (0.95-1.23)	0.230	1.07 (0.93-1.24)	0.500	0.96 (0.81-1.13)	0.800
Phosphate (mmol/L)							
Q1 (0.43~1.05)	524/87250	Ref.		Ref.		Ref.	
Q2 (1.05~1.16)	487/87046	1.03 (0.91-1.16)	0.680	0.97 (0.84-1.13)	0.710	0.95 (0.81-1.12)	0.550
Q3 (1.16~1.27)	479/87213	1.11 (0.97-1.26)	0.120	1.09 (0.94-1.27)	0.240	1.07 (0.91-1.26)	0.390
Q4 (1.27~4.70)	472/86584	1.21 (1.07-1.38)	0.004	1.07 (0.91-1.25)	0.380	1.04 (0.88-1.23)	0.640

Calcium–phosphate products ($mmol^2/L^2$)							
Q1 (0.96~2.48)	525/87023	Ref.		Ref.		Ref.	
Q2 (2.48~2.75)	504/87024	1.06 (0.93-1.20)	0.330	1.03 (0.89-1.19)	0.660	1.00 (0.85-1.17)	>0.99
Q3 (2.75~3.03)	448/87025	1.03 (0.90-1.18)	0.620	0.96 (0.82-1.11)	0.570	0.95 (0.80-1.12)	0.510
Q4 (3.03~9.33)	485/87021	1.24 (1.09-1.41)	0.001	1.13 (0.96-1.31)	0.130	1.06 (0.89-1.25)	0.510
Vitamin D (nmol/L)							
Q1 (10.0~32.4)	561/87455	Ref.		Ref.		Ref.	
Q2 (32.4~46.8)	529/86955	0.88 (0.77-0.99)	0.037	0.87 (0.75-1.00)	0.055	0.85 (0.72-1.00)	0.043
Q3 (46.8~62.3)	456/86877	0.73 (0.64-0.83)	<0.001	0.77 (0.66-0.90)	<0.001	0.77 (0.65-0.91)	0.002
Q4 (62.3~340.0)	414/86721	0.65 (0.57-0.74)	<0.001	0.66 (0.56-0.78)	<0.001	0.68 (0.57-0.81)	<0.001

Abbreviations: Ref, reference group; BMI, body mass index; CI, confidence interval; eGFR, estimated glomerular filtration rate; HbA1c, glycated hemoglobin (Hemoglobin A1c); HDL, high-density lipoprotein cholesterol; HbA1c, glycated hemoglobin (Hemoglobin A1c); HR, hazard ratio; SBP, systolic blood pressure; WC, waist circumference.

Model 1: adjusted for age, sex, Townsend deprivation index, and ethnicity;

Model 2: additionally adjusted for smoking status, alcohol consumption, sleep duration, fruit and vegetable intake, processed meat intake, red meat intake, physical activity level, and total sedentary time;

Model 3: additionally adjusted for HDL-cholesterol concentration, total cholesterol concentration, SBP, BMI, WC, HbA1c, eGFR, antihypertensive medication use, cholesterol-lowering medication, aspirin, insulin, and number of long-term conditions.

Table S6 Sensitivity analysis of serum calcium, phosphate, calcium–phosphate products, and vitamin D levels and incident arrhythmias

Serum mineral levels	Sensitivity analysis 1	P value	Sensitivity analysis 2	P value
	HR (95% CI)		HR (95% CI)	
**All cardiac arrhythmias**				
Calcium (mmol/L)				
Q1 (1.19~2.32)	Ref.		Ref.	
Q2 (2.32~2.37)	0.94 (0.90-0.97)	<0.001	0.94 (0.90-0.97)	0.001
Q3 (2.37~2.43)	0.92 (0.88-0.96)	<0.001	0.92 (0.89-0.96)	<0.001
Q4 (2.43~3.61)	0.91 (0.87-0.95)	<0.001	0.92 (0.88-0.95)	<0.001
Phosphate (mmol/L)				
Q1 (0.43~1.05)	Ref.		Ref.	
Q2 (1.05~1.16)	1.03 (0.99-1.07)	0.100	1.03 (0.99-1.07)	0.110
Q3 (1.16~1.27)	1.05 (1.01-1.09)	0.008	1.05 (1.01-1.10)	0.018
Q4 (1.27~4.70)	1.11 (1.06-1.16)	<0.001	1.11 (1.07-1.16)	<0.001
Calcium–phosphate products ($mmol^2/L^2$)				
Q1 (0.96~2.48)	Ref.		Ref.	
Q2 (2.48~2.75)	1.03 (0.99-1.07)	0.101	1.03 (0.99-1.07)	0.120
Q3 (2.75~3.03)	1.05 (1.01-1.09)	0.006	1.06 (1.02-1.10)	0.019
Q4 (3.03~9.33)	1.08 (1.04-1.13)	0.000	1.09 (1.04-1.13)	<0.001
Vitamin D (nmol/L)				
Q1 (10.0~32.4)	Ref.		Ref.	
Q2 (32.4~46.8)	0.95 (0.90-0.99)	0.004	0.94 (0.90-0.98)	0.015
Q3 (46.8~62.3)	0.96 (0.91-1.00)	0.018	0.95 (0.91-0.99)	0.032
Q4 (62.3~340.0)	0.96 (0.92-1.01)	0.045	0.96 (0.92-1.00)	0.100
**Atrial Fibrillation**				
Calcium (mmol/L)				
Q1 (1.19~2.32)	Ref.		Ref.	
Q2 (2.32~2.37)	0.92 (0.87-0.97)	<0.001	0.92 (0.88-0.96)	<0.001
Q3 (2.37~2.43)	0.90 (0.85-0.94)	<0.001	0.90 (0.86-0.94)	<0.001
Q4 (2.43~3.61)	0.89 (0.84-0.93)	<0.001	0.89 (0.85-0.93)	<0.001
Phosphate (mmol/L)				
Q1 (0.43~1.05)	Ref.		Ref.	
Q2 (1.05~1.16)	1.05 (0.99-1.10)	0.117	1.04 (0.99-1.09)	0.069
Q3 (1.16~1.27)	1.07 (1.02-1.13)	0.006	1.07 (1.02-1.12)	0.006
Q4 (1.27~4.70)	1.16 (1.1-01.22)	<0.001	1.16 (1.10-1.22)	<0.001
Calcium–phosphate products ($mmol^2/L^2$)				
Q1 (0.96~2.48)	Ref.		Ref.	
Q2 (2.48~2.75)	1.03 (0.98-1.08)	0.275	1.03 (0.98-1.08)	0.250
Q3 (2.75~3.03)	1.07 (1.02-1.13)	0.005	1.07 (1.02-1.13)	0.007
Q4 (3.03~9.33)	1.11 (1.05-1.17)	<0.001	1.11 (1.05-1.16)	<0.001
Vitamin D (nmol/L)	Ref.		Ref.	
Q1 (10.0~32.4)	0.95 (0.90-1.00)	0.023	0.94 (0.90-0.99)	0.073

Q2 (32.4~46.8)	0.96 (0.90-1.01)	0.111	0.96 (0.91-1.01)	0.100
Q3 (46.8~62.3)	1 (0.95-1.06)	0.986	1.00 (0.95-1.05)	0.910
**Other arrhythmias**				
Calcium (mmol/L)				
Q1 (1.19~2.32)	Ref.		Ref.	
Q2 (2.32~2.37)	0.95 (0.90-1.01)	0.096	0.96 (0.91-1.01)	0.078
Q3 (2.37~2.43)	0.94 (0.88-0.99)	0.039	0.94 (0.89-1.00)	0.027
Q4 (2.43~3.61)	0.95 (0.89-1.00)	0.101	0.95 (0.90-1.01)	0.059
Phosphate (mmol/L)				
Q1 (0.43~1.05)	Ref.		Ref.	
Q2 (1.05~1.16)	1.02 (0.96-1.08)	0.375	1.02 (0.97-1.08)	0.520
Q3 (1.16~1.27)	1.06 (0.99-1.12)	0.028	1.06 (1.01-1.12)	0.062
Q4 (1.27~4.70)	1.08 (1.02-1.14)	0.005	1.08 (1.03-1.15)	0.012
Calcium–phosphate products ($mmol^2/L^2$)				
Q1 (0.96~2.48)	Ref.		Ref.	
Q2 (2.48~2.75)	1.04 (0.98-1.09)	0.214	1.03 (0.98-1.09)	0.210
Q3 (2.75~3.03)	1.06 (1.00-1.12)	0.023	1.06 (1.01-1.12)	0.042
Q4 (3.03~9.33)	1.08 (1.01-1.14)	0.006	1.08 (1.02-1.15)	0.015
Vitamin D (nmol/L)				
Q1 (10.0~32.4)	Ref.		Ref.	
Q2 (32.4~46.8)	0.95 (0.90-1.01)	0.057	0.95 (0.90-1.00)	0.110
Q3 (46.8~62.3)	0.95 (0.90-1.01)	0.076	0.95 (0.90-1.01)	0.120
Q4 (62.3~340.0)	0.92 (0.86-0.97)	0.002	0.91 (0.86-0.96)	0.004

Abbreviations: Ref, reference group; BMI, body mass index; CI, confidence interval; eGFR, estimated glomerular filtration rate; HDL, high-density lipoprotein cholesterol; HbA1c, glycated hemoglobin (Hemoglobin A1c); HR, hazard ratio; SBP, systolic blood pressure; WC, waist circumference.

Model adjusted for age, sex, Townsend deprivation index, ethnicity, smoking status, alcohol consumption, sleep duration, fruit and vegetable intake, processed meat intake, red meat intake, physical activity level, and total sedentary time, HDL-cholesterol concentration, total cholesterol concentration, SBP, BMI, WC, HbA1c, eGFR, antihypertensive medication use, cholesterol-lowering medication, aspirin, insulin, and number of long-term conditions.

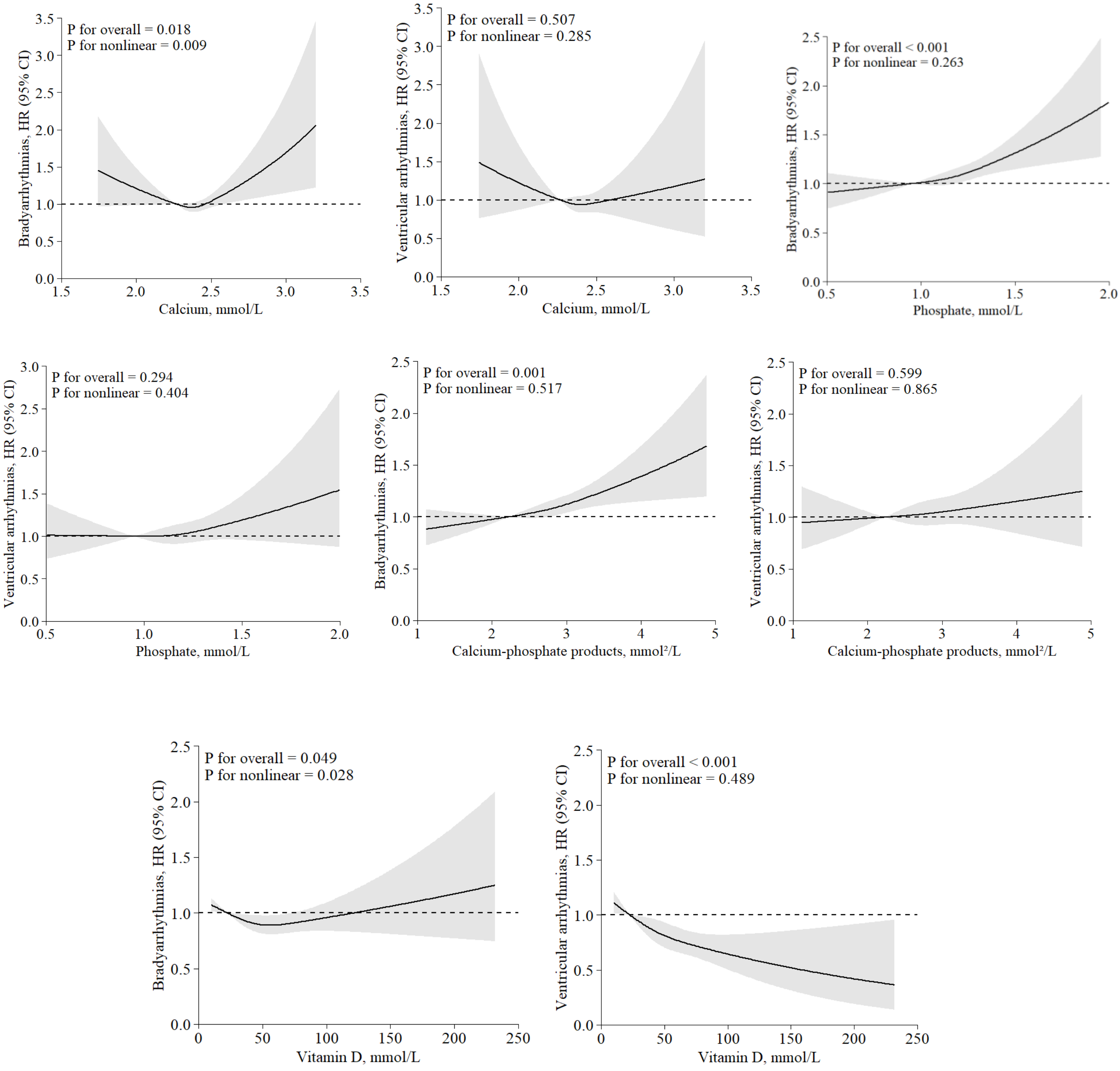

Figure S1 Dose-response association between serum calcium, phosphate, calcium–phosphate products, and vitamin D levels and bradyarrhythmia and ventricular arrhythmias

Model adjusted for age, sex, Townsend deprivation index, ethnicity, smoking status, alcohol consumption, sleep duration, fruit and vegetable intake, processed meat intake, red meat intake, physical activity level, and total sedentary time, HDL-cholesterol concentration, total cholesterol concentration, SBP, BMI, WC, HbA1c, eGFR,

antihypertensive medication use, cholesterol-lowering medication, aspirin, insulin, and number of long-term conditions.

BMI, body mass index; CI, confidence interval; eGFR, estimated glomerular filtration rate; HDL, high-density lipoprotein cholesterol; HbA1c, glycated hemoglobin (Hemoglobin A1c); HR, hazard ratio; SBP, systolic blood pressure; WC, waist circumference.
